# Neurological Diseases Define the Cytokine Profile in CFS during SARS-CoV-2 Infection in Highly Ill Patients

**DOI:** 10.3390/tropicalmed8060290

**Published:** 2023-05-25

**Authors:** Lucía Angélica Méndez-García, Helena Solleiro-Villavicencio, Sebastián Guartazaca-Guerrero, Jahir Rodríguez-Morales, José Damián Carrillo-Ruiz

**Affiliations:** 1 Laboratory of Immunometabolism, Research Division, General Hospital of Mexico “Dr. Eduardo Liceaga”, Mexico City 06720, Mexico; lucia.mendez@salud.gob.mx; 2 Posgrado en Ciencias Genómicas, Universidad Autónoma de la Ciudad de México, Mexico City 03100, Mexico; helena.solleiro@uacm.edu.mx; 3 Neurology and Neurosurgery Unit, General Hospital of Mexico “Dr. Eduardo Liceaga”, Mexico City 06720, Mexico; 4 Coordination of Neuroscience, Faculty of Psychology, Mexico Anahuac University, Mexico City 52786, Mexico

**Keywords:** neuroinflammation, COVID-19, SARS-CoV-2, cerebrospinal fluid

## Abstract

Neuroinflammation is critical in developing and progressing neurological diseases. The underlying pro-inflammatory cytokine expression combined with additional mechanisms in the neuropathology, such as oxidative stress, brain–blood barrier damage, and endothelial dysfunction, could contribute to the susceptibility to developing severe COVID-19. The physiopathology of SARS-CoV-2 and other human coronaviruses (H-CoVs) has not been completely understood; however, they have all been linked to a disproportionated response of the immune system, particularly an exacerbated cytokine production and the dysregulation of total cell counts. In this article, based on the compilation of studies reported by our working group regarding COVID-19 and neurological diseases, we propose that the inflammation observed in the central nervous system, through a CSF analysis, could be conditioned by neurological disease(s) and enhanced by COVID-19. Therefore, it is necessary to determine the cytokine profile in different neurological disorders to propose adequate treatments and avoid severe forms of the disease in these patients.

## 1. Introduction

COVID-19 is caused by the severe acute respiratory syndrome coronavirus 2 (SARS-CoV-2). In addition to COVID-19 primarily affecting the respiratory system, several neurologic symptoms associated with the disease have been reported. These symptoms can be mild and non-life threatening (e.g., anosmia, headache, dizziness, etc.); however, some are critical and life-threatening (e.g., stroke or seizure) [1].

Several studies suggest that the neurological symptoms of COVID-19 result from the inflammatory response due to SARS-CoV-2 infection, even though the virus does not reside within the central nervous system (CNS). Nevertheless, new evidence confirms that the SARS-CoV-2 virus may also enter the brain through different routes [2]: (1) By retrograde axonal transport and trans-synaptic viral spreading from the olfactory epithelium along the olfactory nerve to the olfactory bulb via the transcribral route. (2) By hematogenous invasion due to blood–brain barrier (BBB) leakage. (3) By brain lymphatic drainage system (“Trojan horse mechanism”) that refers to the capacity of monocytes and macrophages to become a viral pool that might diffuse the virus toward the CNS. (4) SARS-CoV-2 can enter the CNS by binding to the angiotensin-converting enzyme 2 (ACE2), which has been reported to be expressed in various brain cells and cerebral parts that lack BBB [2,3,4].

Either by the direct invasion of the virus or by the influence of the cytokine storm, COVID-19 worsens pre-existing neurological conditions, probably by exacerbating the pre-existing neuroinflammation.

There is evidence that neuroinflammation is a critical factor in developing neurological diseases. In this sense, several studies have been carried out to determine the profile of cytokines in the CSF of these patients with different neuropathologies and the correlation of the CSF levels of both pro- and anti-inflammatory molecules with the severity of the disease. Alzheimer’s disease (AD) is extensively researched. Studies have found that specific cytokines, such as eotaxin, interleukin [IL]-1ra, IL-4, IL-7, IL-8, IL-9, IL-10, IL-15, monocyte chemotactic protein 1 (MCP-1), and tumor necrosis factor-alpha (TNF-α), are overexpressed in cerebrospinal fluid (CSF) in comparison to the control group [3]. Furthermore, an inverse correlation has been demonstrated between some cytokines (e.g., IL-1β, IL-4, IL-6, IL-9, and IL-17A) with the disease progression, highlighting the importance of both the pro-inflammatory and anti-inflammatory states in the development and progression of neurological diseases [5]. Apart from neuroinflammation, other mechanisms in neuropathologies like oxidative stress, BBB damage, and endothelial dysfunction may also increase the vulnerability to severe COVID-19.

In addition to the underlying neuroinflammation and the mechanisms that make patients with neurological diseases more prone to severe COVID-19, various studies have contributed to understanding the immune system response due to infection by SARS-CoV-2. COVID-19 symptoms vary greatly, even among individuals. The differences in the abundance and frequency of the immune cells’ populations have been largely described among different groups of patients. Thus, patients with severe COVID-19 disease have been shown to have increased populations of neutrophils, eosinophils, and monocytic myeloid-derived suppressor cells compared to patients with mild COVID-19 [6]. On the contrary, it has been observed that the plasmatic dendritic cell population (pDCs) decreases in patients with severe COVID-19, which could impact the IFN-I/λ antiviral response of these patients [7]. A hallmark of patients with severe COVID-19 is lymphopenia [8], characterized by a dramatic decrease in CD4+ and CD8+ T cells [8], CD8^+^ mucosal-associated invariant T (MAIT), and γδ T cells [9]. In addition to the cell numbers, it has been observed that there is an increase in cell activation markers of NK cells and a reduction of markers such as HLA-DR and CD86 on monocytes in patients with a severe form of the disease [10].

Given the information provided, the concern is whether inflammation in the cerebrospinal fluid of individuals with neurological disorders may worsen after contracting a SARS-CoV-2 infection. To identify treatment targets and prevent the exacerbation of neurological diseases triggered by viral infections, it is crucial to understand the cytokine profiles and CSF biomarkers in affected patients.

## 2. Relevant Section

At the initial stage of the pandemic, we made a follow-up to four patients with COVID-19 (before vaccination) with previous diagnoses of cerebrovascular disease (CVD) or brain tumors. These patients arrived at the General Hospital of Mexico due to neurological symptoms; however, after a nasal swab test, they were positive for SARS-CoV-2. Moreover, during surgery, by the ventricular shunt, a cerebrospinal fluid (CSF) sample was taken and analyzed to determine the presence of the virus within the CNS, the inflammatory profile, and biochemical parameter values [11,12]. As previously reported, for Cases 1 and 2, the CSF cytokine analysis was performed using the Human Cytokine Magnetic 25-Plex Panel (Life Technologies, Frederick, MD, USA) following the manufacturer’s instructions [11]. Results were analyzed with the MILLIPLEXTM Analyst 5.1 Flex software. From the 25-cytokine panel, both patients showed higher levels of IL-6, IL-12, eotaxin, IL-17, MIP-1a, GMC-S, MIP-1b, MIG, IL-15, IFN-γ, IFN-α, TNF-α, and IL-7, but within this panel of cytokines, MCP-1 and IL-8 presented an overwhelming overexpression compared with those reported in the literature. Instead, the CSF cytokine profiles of Case 3 and Case 4 were determined by qPCR (CFX96TM Real-Time System, BIO-RAD, Hong Kong, China) with SYBR Green Master Mix (Jena Bioscience, Jena Germany, Jena, Germany, Cat number PCR-372L) using specific primers for 25 cytokines designed using the Primer-BLAST software from the National Center for Biotechnology Information, U.S. National Library of Medicine [12]. The expression of the cytokines was normalized using the housekeeping gene control 18 s and reported as 2∆∆Ct. We used a control CSF sample taken by a ventricular shunt from a patient with solitary fibrous tumor (SFT) named hemangiopericytoma. Cytokines such as IFN-α, IL-4, and TGF-β showed a striking overexpression compared to the control (56–97-fold changes). Moreover, the cytokines IL-12, IL-13, and IL-2 overexpressed above 100-fold-change in Case 3 and above 60-fold-change in Case 4.

Table 1 summarizes the main respiratory and neurological symptoms, the neurological pathologies, the central cytokines found to be overexpressed in the CSF, and the elevated biochemical parameters in each patient.

**Figure 1:**
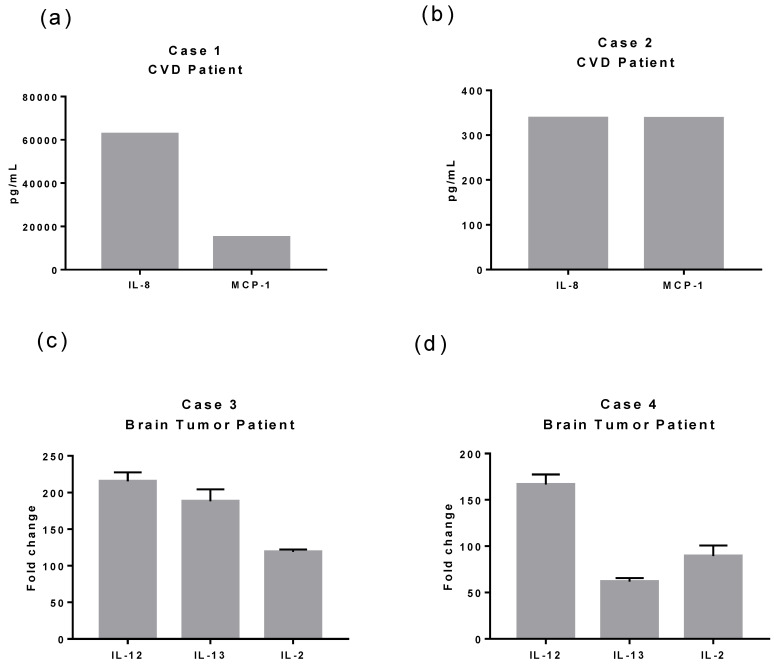
Overexpressed cytokines in CSF of patients with CVD or brain tumor and COVID-19. We analyzed the protein cytokine profile (25 cytokines) in Cases 1 and 2. Meanwhile, we evaluated the cytokine expression using quantitative RT-PCR in Cases 3 and 4 (25 cytokines). (**a**) Case 1 presented and exacerbated expression of IL-8 (>60,000 pg/mL) and MCP-1 (15,000 pg/mL) compared to Case 2 (**b**) (IL-8 300 pg/mL and MCP-1 337.51 pg/mL) and compared with reported physiological levels (IL-8 21.40 ± 7.96 pg/mL [13] and MCP-1 160.95 pg/mL [14]) and COVID-19 patients with any pre-existing neurological condition (IL-8 83.2 (68.8–161.1) pg/mL and MCP-1 1216 (873.4–3932.3) [15]). (**c**) Case 3 had elevated expression levels of IL-12 (approximately 215-fold changes), IL-13 (188-fold changes), and IL-2 (119-fold differences). (**d**) Case 4 presented elevated expression of IL-12 (166-fold changes), IL-2 (89-fold differences), and IL-13 (61-fold changes). Case 3 and Case 4-fold changes were calculated using a CSF sample control from a patient with SFT. Case 3 was the only positive for SARS-CoV-2 mRNA in the CSF.

At the systemic level, all patients with pre-existing neurological disease and COVID-19 showed abnormalities in the total immune cell count: leukocytosis and neutrophilia. It was observed that three of the four patients had monocytopenia and lymphopenia. As previously reported, no viral particles were found in the CNS in the two patients with CVD. Moreover, and as mentioned earlier, CSF showed a cytokine profile enriched mainly by IL-8 and MCP-1 (Figure 1A,B). On the other hand, the patients with brain tumors had increased levels of IL-12, IL-2, and IL-13 in their cytokine profile (Figure 1C,D). A striking result was that the concentration of these cytokines was significantly higher (Figure 1C) in the patient with a high albumin Q index, suggestive of damage in the BBB; in addition, SARS-CoV-2 mRNA was found in the CSF of this patient.

**Figure 2:**
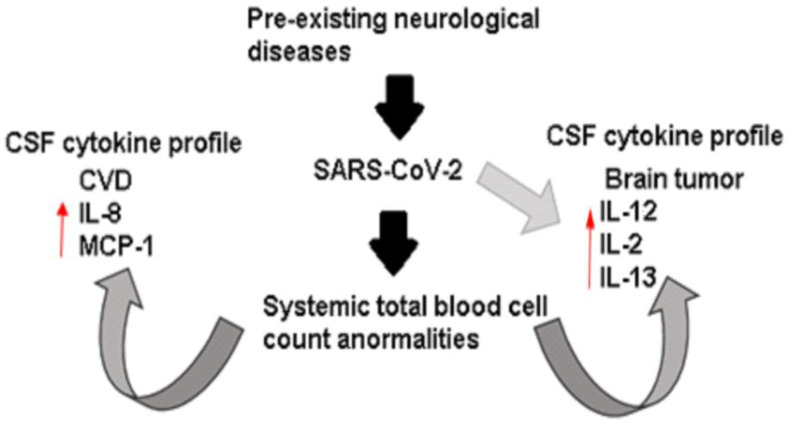
Immunological dysregulation in extremely ill patients with neurological disease and COVID-19. Systemically, the patients showed abnormal counts of total blood cells characterized by leukocytosis, neutrophilia, and lymphopenia. However, the cytokine profile presented in CSF seems conditioned by neurological diseases and exacerbated by the SARS-CoV-2 infection.

In summary, at a systemic level, we found that these four patients had the same leukocytic dysregulations. However, we discovered notable variations in the cytokine profile of the CSF, depending on whether the patient had a pre-existing neurological condition such as cardiovascular disease or tumor, further intensified by the presence of SARS-CoV-2 infection. Therefore, in this opinion article, we hypothesize that the cytokine profile in CSF of COVID-19 patients may be conditioned by the pre-existing neurological disease and exacerbated by the viral infection (Figure 2).

## 3. Discussion

COVID-19, caused by SARS-CoV-2, is usually a mild respiratory infection; however, some patients can develop pneumonia and acute respiratory distress syndrome (ARDS). ARDS is the leading cause of death due to COVID-19, characterized by high levels of pro-inflammatory cytokines (IL-6, IL-17, IL-1β, and TNF-α) and immune cell hyperactivation, a state called “cytokine storm” that may lead to multiorgan failure [16]. COVID-19 patients have reported both central and peripheral nervous system manifestations. Certain authors have also noted a correlation between the intensity of cytokine storms and the severity of neurological manifestations [16].

Consistent with our results, several groups have reported that high numbers of neutrophils and low circulating lymphocytes characterize COVID-19-associated cytokine storms. Furthermore, monocytopenia is mainly observed in patients with severe infection and is a feature our patients had [17].

In the context of brain tumors, it is known that the stromal contribution of the tumoral microenvironment (TME) and the role of immune cells are critical regulators of cancer progression in the brain [18]. It has been reported that the most abundant cytokines in meningiomas are IFN-γ, TNF-α, and TGF-β [18]. Moreover, in the serum of patients with glioblastoma (the most typical parenchymal brain tumor), it has been reported that there is a significant overexpression of IL-6, IL-1β, TNF-α, IL-10, VEGF, FGF-2, IL-8, IL-2, and GM-CSF [19]. In addition, it has been observed that the levels of these cytokines are strongly correlated with tumor grade, proliferation markers, and clinical aggressiveness in glioblastomas [19]. Accordingly, both patients with brain tumors had a CSF cytokine profile with high IL-12, IL-13, and IL-2 concentrations.

On the other hand, some authors have reported increased levels of serum inflammatory markers after a spontaneous intracerebral hemorrhage (ICH). These levels correlate with both radiographic parameters of brain injury and clinical outcomes [20]. In addition, cytokines in ICH models have been described as mediators of increased vascular permeability, inducing edema formation, migration of leukocytes into the brain, and causing cytotoxic effects and activation of other bioactive compounds [20]. Ziai et al. have recently provided information on the inflammatory cytokine profile in patients with ICH, revealing higher levels of IL-1β, IL-6, IL-10, TNF-α, and CCL2 in their CSF. However, these cytokine levels tend to decrease over the next ten days [20]. Our research [11,12] discovered that patients with ICH have higher levels of IL-8 and MCP-1 in their cerebrospinal fluid. This indicates an increase in inflammatory and chemoattractant substances, different from previously reported but still relevant to this medical condition.

It has recently been proposed that persons with pre-existing neurological comorbidity showed an OR of 2.3 suffering from severe COVID-19 and a 2.4-fold higher mortality risk in patients with cerebrovascular or cardiovascular disease [21,22]. In that sense, the patients we show with a particular pro-inflammatory cytokine profile also seem more susceptible to severe clinical forms of COVID-19. It is important to note that patients with a higher cytokine expression, regardless of their profile, eventually died from the disease. This was observed in case 1, whose Glasgow Coma Scale (GCS) was 6 points, and case 3, which had viral particles in their cerebrospinal fluid (CSF) and initially had a GCS of 13 points, which gradually worsened due to the viral infection. Unlike cases 1 and 3, cases 2 and 4 had lower levels of cytokines and an initial GCS between 13 and 14. Therefore, they were discharged because of improvement. Accordingly, it has been reported that high levels of cytokine and chemokine in the cerebrospinal fluid (CSF) are linked to an increased risk of mortality [23]. This information emphasizes the significance of analyzing the cytokine levels in cerebrospinal fluid when evaluating COVID-19 patients with pre-existing neurological conditions.

Our data show that viral infections can worsen the existing cytokine profile in the brain. Additionally, our findings align with those of other researchers, indicating that the presence of SARS-CoV-2 RNA is not linked to the severity of neuropathological changes.

## 4. Conclusions and Future Directions

Although our data is highly informative, we must exercise caution when concluding. It is essential to remember that the wide range of neurological conditions means a significant amount of variation between different medical facilities and pathological situations. As a result, it is not feasible to establish a definitive cut-off level for any of the molecular factors involved [12]. Hence, more accurate pathological evidence is warranted. Our data reveal that patients with neurological diseases have a higher risk of severe COVID-19 due to their pre-existing neuroinflammatory profile. This highlights the need for careful health surveillance. Furthermore, the data raises new inquiries regarding the possible utilization of anti-cytokine and immune-modulating therapies for COVID-19 patients with underlying diseases. Examining the patient’s background CSF cytokine profile may be advantageous to tailor the therapeutic approach for those with the infection.

## Figures and Tables

**Table 1:** Clinical, anthropometric, biochemical, and immunologic features of patients with neurological diseases and COVID-19.

	Gender	Age (Years)	Pre-Existing Neurological Disease	Respiratory/Neurological Symptoms Due to COVID-19	Neurological Symptoms at Arrival to the Hospital	Presence of SARS-CoV-2 Nasopharyngeal/CSF	Deregulated Biochemical Parameters	Immunological Parameters	Cytokine Profile in CSF	
Case 1	Female	54	CVD (acute intra-axial hemorrhage)	Without symptoms	-Moderate headache -Balance impairment -Weakness -Loss of consciousness	Positive/Negative	Dimer-D (14,760 Ug/L) Glucose (161 mg/dL) Total cholesterol (236 mg/dL)	Leukocytosis (12.10 × 10^3^/mm^3^) Lymphopenia (0.70 × 10^3^/ mm^3^) Neutrophilia (11.30 × 10^3^/ mm^3^) Monocytopenia (0.1 × 10^3^/ mm^3^)	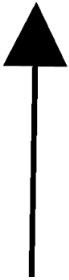	IL-8 MCP-1 IFN-α
Case 2	Male	45	CVD (diffuse subarachnoid hemorrhage)	Without symptoms	-Seizure -Psychomotor agitation -Disorientation	Positive/Negative	Dimer-D (4304 Ug/L) Fibrinogen (562 mg/dL)	Leukocytosis (15.70 × 10^3^/ mm^3^) Lymphopenia (0.90 × 10^3^/ mm^3^) Neutrophilia (14.20 × 10^3^/ mm^3^)	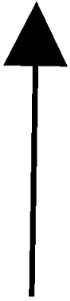	IL-8 MCP-1 IFN-α
Case 3	Female	43	Brain tumor (petroclival meningioma)	Pneumonia	-Severe headache -Drowsiness	Positive/Positive	Triglycerides (372 mg/dL) Glucose (110 mg/dL) ALT (70 U/L) Q-albumin (7.3 × 10^−3^ g/dL)	Leukocytosis (12.40 × 10^3^/ mm^3^) Neutrophilia (9.10 × 10^3^/ mm^3^) Monocytopenia (0.2 × 10^3^/ mm^3^)	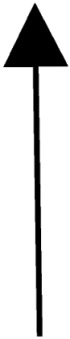	IL-12 IL-2 IL-13
Case 4	Male	43	Brain tumor (in the fourth ventricle)	Anosmia Peripheral oxygen saturation of 89% Neurological impairment Pneumonia	-Intense headache -Syncope -Gait abnormalities -Decreased alertness	Positive/Negative	ALT (79 U/L)	Leukocytosis (12.40 × 10^3^/ mm^3^) Neutrophilia (14.20 × 10^3^/ mm^3^) Lymphopenia (0.90 × 10^3^/ mm^3^) Monocytopenia (0.1 × 10^3^/ mm^3^)	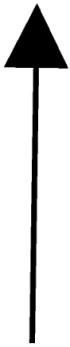	IL-12 IL-2 IL-13

Abbreviations: cerebrovascular disease (CVD), alanine transaminase (ALT). Up arrow represents increased concentrations of the indicated cytokines.

## Data Availability

The articles on which we base ourselves to write this opinion are published and can be consulted at the following links: https://pubmed.ncbi.nlm.nih.gov/34230225/; https://pubmed.ncbi.nlm.nih.gov/36050226/ (Accessed on 23 May 20223).
